# Mapping ethical, legal, and social implications (ELSI) of preimplantation genetic testing (PGT)

**DOI:** 10.1007/s10815-024-03076-y

**Published:** 2024-03-21

**Authors:** Ido Alon, Ilona Bussod, Vardit Ravitsky

**Affiliations:** 1 Department of Development Economics, Autonomous University of Madrid, Madrid, Spain; 2 University of Montreal, Montreal, Canada

**Keywords:** Ethical, Legal, And Social Implications (ELSI), Preimplantation Genetic Diagnosis, Geographic distribution of research, Gaps in research, Mapping

## Abstract

**Purpose:**

Preimplantation Genetic Testing (PGT) has attracted considerable ethical, legal, and social scrutiny, but academic debate often fails to reflect clinical realities.

**Methods:**

Addressing this disconnect, a review of 506 articles from 1999 to 2019 across humanities and social sciences was conducted to synthesize the Ethical, Legal, and Social Implications (ELSI) of PGT. This review mined PubMed, WoS, and Scopus databases, using both MeSH terms and keywords to map out the research terrain.

**Results:**

The findings reveal a tenfold increase in global research output on PGT’s ELSI from 1999 to 2019, signifying rising interest and concern. Despite heightened theoretical discourse on selecting “optimal” offspring, such practices were scarcely reported in clinical environments. Conversely, critical issues like PGT funding and familial impacts remain underexplored. Notably, 86% of the ELSI literature originates from just 12 countries, pointing to a research concentration.

**Conclusion:**

This review underscores an urgent need for ELSI research to align more closely with clinical practice, promoting collaborations among ethicists, clinicians, policymakers, and economists. Such efforts are essential for grounding debates in practical relevance, ultimately steering PGT towards ethical integrity, societal acceptance, and equitable access, aiming to harmonize PGT research with real-world clinical concerns, enhancing the relevance and impact of future ethical discussions.

## Introduction

Genetic selection of human embryos has been raising questions concerning Ethical, Legal, and Social Implications (ELSI) long before Assisted Reproductive Technologies (ART) became medically feasible. These ELSI assessments are influenced by cross-cultural differences and value-based perspectives and have an impact on the evolution of clinical practices and regulations, concerning which genetic conditions may be diagnosed (i.e., monogenetic or multifactorial, of early or late onset, with complete or reduced penetrance, curable or non-curable), applications of PGT against aneuploidies, and practices for non-medical reasons [1–9].

Preimplantation Genetic Testing (PGT) is a complement to ART that has been practiced since 1990. It refers to the genetic analysis of a biopsy removed from an embryo and used to select against various genetic conditions and characteristics. Specifically, PGT-M (monogenic disorders) targets single-gene disorders, which are caused by mutations in a single gene, affecting traits or causing diseases such as cystic fibrosis. PGT-SR (structural rearrangements) addresses hereditary chromosome abnormalities, where the structure or number of chromosomes is altered, potentially leading to conditions like Down syndrome. PGT-P (polygenic disorders) aims to reduce the risk of diseases influenced by multiple genes and environmental factors, such as diabetes, cancerous, and cardiovascular diseases. Additionally, sex selection (SS) is used for medical and non-medical reasons, often to prevent sex-linked genetic diseases. In recent years, PGT-A (aneuploidy screening) has become common to select against aneuploidies, conditions where the number of chromosomes is not the standard 46, helping to increase implantation rates, reduce the risk of miscarriage, and avoid chromosomal abnormalities [10–13]. Less frequently, PGT with human leukocyte antigen (HLA)-matching is practiced to select embryos that are both free of disease-related mutations and HLA identical to sibling, potentially serving as a compatible future donor for an affected sibling [14].

PGT is applied to an increasing proportion of ART cycles worldwide, with 4.8% of ART cycles in Europe involved PGT in 2018 according to the latest ESHRE report (ESHRE, 2022). In the U.S., it had grown from 5 to 22% of ART cycles between 2015 and 2016, and from 32 to 44% of embryo transfers^1^ between 2017 and 2019, as it was the primary reason to carry out 11–15% of the ART cycles (CDC, 2017-2018, 2019-2021). Overall, the representation of PGT-M, PGT-SRT, and PGT-P applied to ART cycles worldwide is much smaller in comparison with PGT-A [15].

In this paper, we review the global academic literature on the ELSI of PGT that was published in English between 1999 and 2019. It includes research of bioethical**,** psychological, sociological, anthropological, legal, and economic perspectives, around topics such as morality and responsibility, concerns about eugenic uses, public knowledge and attitudes, patient’s experience, social implications, legal restrictions, religious perceptions, availability, and access to treatment. This literature is influenced by cultural perceptions of, and attitudes towards embryo status, family structures, parent–child relationship, and disability. It has an impact on the regulations of PGT which vary between countries and different cultural contexts, and define which genetic conditions are diagnosed, which services are funded, and which non-medical uses are allowed (Klitzman, 2009; [2, 8, 16–20]).

Previous reviews analyzed various aspects of PGT, such as technological trends in the past, present, and future [21, 22]; regulatory differences between countries [23, 24], social dimensions [25], ethical considerations [26], and patients knowledge and perceptions [27], among other approaches.

We extracted a corpus of literature of 506 articles dealing with the ELSI of PGT from PubMed, Web of Science, and Scopus, spanning the years 1999 to 2019. Our analysis provides a unique review of the research agenda, design, and geographical distribution, aiming to identify gaps in research and unveil areas that receive disproportionate attention. Ultimately, this review underscores the divergence between global academic discussions on PGT and the real-world clinical challenges, underlining crucial issues of reproductive justice that often remain underexplored.

## Methods^2^

### Design

The corpus of literature of ELSI of PGT was extracted together with a larger corpus concerning the ELSI of ART. We designed inclusion criteria to select articles dealing with ELSI of ART and exclusion criteria to exclude articles dealing entirely with clinical and medical matters, as described in Appendix 1 (Table 3).

### Collection

The corpus was collected from the online databases PubMed, Web of Science (WoS), and Scopus. Following a keywords frequency analysis, three groups of Medical Subject Headings (MeSH) terms were selected, as shown in Appendix 2 (Table 4). Group A included ART terms and Group B included terms indicating a relation to ELSI across disciplines within humanities and social sciences. Group C was formed to exclude irrelevant articles. We aimed to find balance between false-positive (inclusion of articles with medical-clinical nature) and false-negative (exclusion of articles concerning social sciences and humanities).

We used the PubMed API [28] to query for articles with “One MeSH-term from group A” AND “One MeSH-term from group B” AND “Humans (MeSH)” AND “1999–2019” NOT “Any MeSH-term from group C.” To begin with, we included all articles that had an abstract in English, regardless of the language of the article.

The PubMed query brought up 11,246 results of which 7003 had a full record of title and abstract in English. Additionally, 159 articles which were queried with no full record from PubMed were imported from Scopus. In total, 7162 articles had a full record.

We dropped abstracts with less than 50 words (259); removed articles if article type included “Clinical Trial,” Controlled Clinical Trial,” “Randomized Controlled Trial,” or “Validation Study” (536); and excluded all journal related to biodiversity (34). Six thousand three hundred thirty-three articles remained; we extracted a list of keywords including their frequencies within the titles and abstracts and divided them into three groups (see Appendix 2 (Table 4)) with similar definition as described above. We queried the WoS and Scopus APIs for articles of which the title, abstract, or keywords had “One term from group A” AND “One term from group B” AND “1999–2019″ NOT “Any term from group C.”

We extracted 14,394 and 12,588 articles from WoS and Scopus, respectively. In addition to the 6333 extracted from PubMed, 33,315 articles were merged from all three databases. Following the removal of duplicates of titles, abstract, and DOI, 17,247 articles remained, of which 14,283 had available title and abstract in English. We repeated the cleaning methods previously applied on the PubMed query (explained above), removed 154 articles due to short abstract (less than 50 words), and 99 from journals of biodiversity. Fourteen thousand and thirty articles remained.

### Cleaning

Two researchers cleaned up the corpus by analyzing the titles and abstracts with an emphasis on the rejection criteria from Appendix 1 (Table 3). We removed 6315 articles and remained with a corpus of 7714 relevant articles of full record of which 1184 were non-English articles, with English abstracts.

The abstracts were processed by merging the title and abstract into one string (“code”), harmonized, tokenized,^3^ and lemmatized. We formed a list of terms to be replaced with acronyms or abbreviations in order to unify the text and allowing us to identify repetitions. Next, we extracted a list of terms by the frequency of “codes” in which they appear, and divided the most frequent technical-medical terms into ART field. For this review, we collected the terms that belong to the field of PGT, as appeared in Table 1. An article was included in the final corpus if one term, associated to PGT, was found in its title, abstract, or keywords. As a result, 1019 articles out of 7714 were selected.

**Table 1:** Words and terms for inclusion

Preimplantation Genetic Testing	_pgd_ • _pgs_ • _pgt_ • embryo select • enhancement • gender select • genetic counsel • genetic select • genetically select • hla matched • hla typing • pre implantation diagnos • pre implantatory exam • preimplantation diag • preimplantation or prenatal screening • preimplantational • preimplantatory • procreative benef • reproductive genetic • saviour sibling • selection of embryo • sex select • whole genome sequencing

**Table 2:**
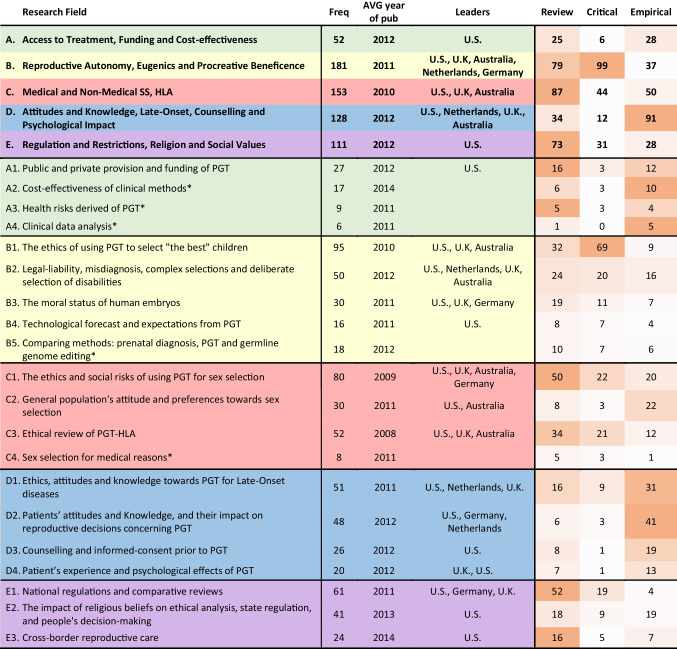
Clusters of research questions.

*The groups A2, A3 A4, B5, and C4 cover questions of semi-clinical character that were captured in our search for ELSI literature. These research niches include a much larger number of articles which were not captured in the corpus due to the exclusion of article dealing entirely with clinical and medical matters.

Subsequently, two researchers filtered the corpus based on the full texts and removed 513 articles (141 were not written in English, 313 were not focused or only marginally focused on PGT, 56 were not available online, 3 were not full-length articles). Finally, 506 articles of full text remained.

### Classification and analysis

Two researchers classified and extracted from each article the following information through content and thematic analysis: Study type (Conceptual, Review, Empirical, etc.), Design (Methods), Objectives (Research questions), and Outcome (Summary of results and conclusions). Additionally, with view of the original author keywords and based on the full texts, the authors extracted uniform keywords to maintain the same contextual meaning for the whole corpus, while removing general-technical keywords (i.e., IVF, PGT, etc.).

For each publication, all obtainable metadata was extracted from WoS, Scopus, and PubMed, merged and unified under one template and the original author keywords were replaced with the uniform ones. The corpus was uploaded to the VOSviewer software tool for constructing and visualizing bibliometric networks, to extract cluster analysis according to co-occurrence of keywords, in which only the keywords that were repeated at least in 9 articles were considered.^4^ The VOSviewer analysis raised 5 clusters of research fields. Considering keywords, in addition to the objectives and outcome extracted from the full texts, we classified the articles into those 5 clusters, dividing each cluster to several relevant groups of research questions (20 in total).

## Results

Between 1999 and 2019, the global research output about ELSI of PGT increased from less than 10 articles per year in 1999–2001 to 20–30 per year in 2005–2017 and to 38 publications in 2018 and 49 in 2019.

### Research framework

**Fig. 1:**
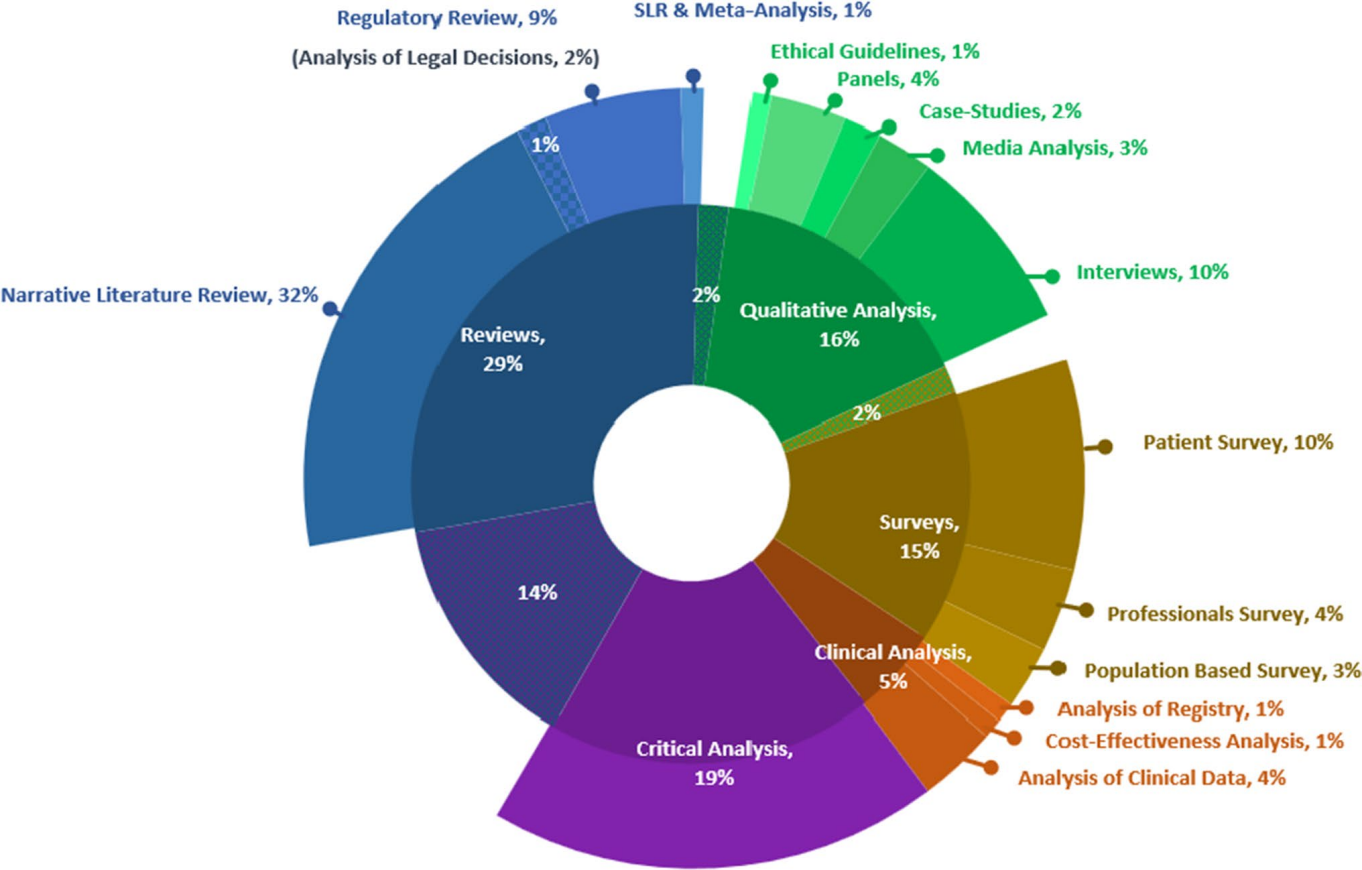
Methods (research design). *The parts with the patterned fill represent overlap of study types

Figure 1 presents study types and methods adopted by researchers. About 35% of the 506 publications were dedicated to empirical research, including qualitative analysis (20%), of which interviews (10%) were the most frequent approach; surveys (17%), mainly with patients (10%); and clinical analysis (5%), mainly of clinical data (4%). Conceptual (critical) analysis represented 33% of the literature. These conceptual papers often go together with review articles (an overlap of 14%). Under reviews (45%), we identified 32% narrative literature review and 9% regulatory review (which is usually done in a narrative manner). Meta-analysis represented only 1% of the literature.

### Research fields

**Fig. 2:**
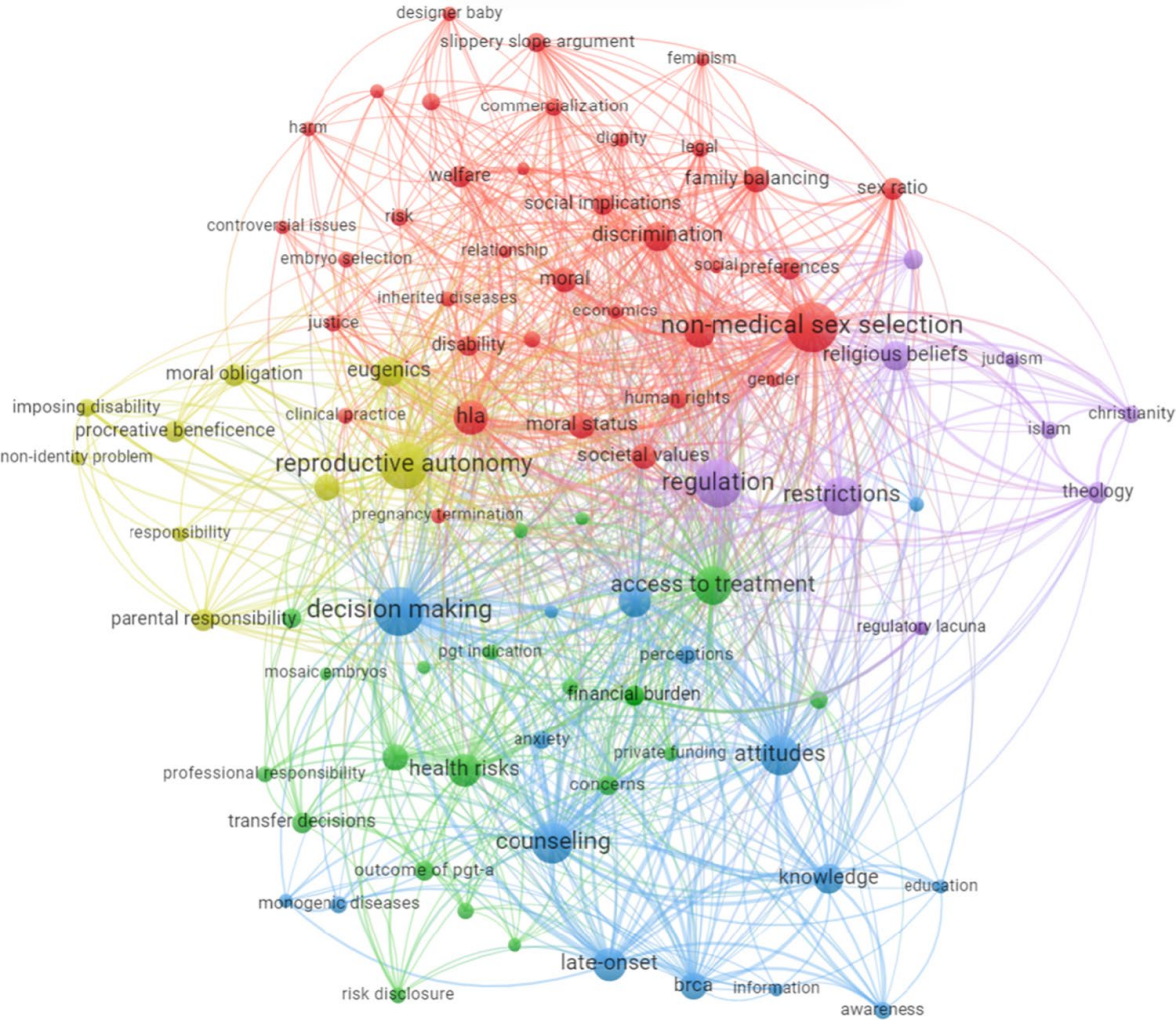
Co-occurrence of keywords (clusters). We invite the reader to consult the interactive version of this figure, available online at: https://app.vosviewer.com/?json=https://drive.google.com/uc?id=12DeLAs61-okICe4nd9JxbyzUM0MSuagi

The results of a VOSviewer analysis, presented in Fig. 2, display five clusters of research fields arising from the corpus. Cluster A (green), elaborated mostly through reviews and empirical research, gathers questions of access, affordability and financial burden, cost-effectiveness, risks, and outcomes of PGT. Cluster B (yellow), mainly handled by critical analysis and reviews, covers questions of reproductive autonomy, enhancement, eugenics, embryo status, procreative beneficence, and parental responsibility. Cluster C (red), strongly approached by review articles, focuses on sex selection in addition to PGT for HLA-typing. Cluster D (blue), with a large share of empirical research, deals with decision-making, counselling, psychological impacts, and PGT for late-onset diseases. Cluster E (purple), approached strongly by review articles, focuses on regulations, restrictions, and religious beliefs.

Each cluster was divided into several groups of research questions as seen in 2. The following is an overview of the major groups:

## Cluster A (blue)—access to treatment, funding, and cost-effectiveness

**A1**. Twenty-seven articles were dealing with public and private funding and provision of PGT. The leading methodological approach was literature review (16 articles, 59%), followed by 12 articles of empirical research (44%). Nine (33%) of these articles were written by authors from the U.S.; others were written by authors from 10 other countries. Articles aimed to report availability, access to, and financial burden of PGT in different countries with a focus on inequality in access to treatment [29–31]. Some articles raised arguments for and against public coverage [32–34]. Others discussed issues as organization of the clinics, counselling and medical referral, rules for reimbursement, and other factors that influence patients’ decision-making and constitute barriers to access [35, 36]. There were only a few examples of genuine cost–benefit analysis of public coverage which compared the cost of funding PGT to the cost of caring for patients suffering from the genetic conditions in question [37, 38].

**A2**. This group consists of 17 articles examining the cost-effectiveness of various clinical methods. Among these, 59% (10 articles) employed empirical research as their foundation. Notably, authors from the U.S. and U.K. contributed to 35% (6 articles) of these papers, while the remaining were authored by researchers across 11 different countries. A recurring theme within these articles centered on the outcomes and cost-effectiveness of PGT-A, with 10 articles specifically addressing this issue. We recognize that, due to our selection criteria, numerous medically focused articles discussing similar topics were not included in our dataset. Over the years, PGT-A outcomes have been a subject of intense debate among scholars.

## Cluster B (yellow)—reproductive autonomy, eugenics, and procreative beneficence

**B1**. The largest group dealt with the ethics of using PGT to select “the best” children. It included 95 articles, 18.8% of the corpus. Sixty-nine (73%) of the articles were conceptual (critical) analysis, and 71 (75%) were written by authors from the U.S. (35), U.K. (22), and Australia (14). The debate surrounding this topic, which dates back long before PGT became an established clinical practice, stems from the association of genetic selection to the eugenics movement of the late nineteenth and early twentieth centuries. The term “eugenics” was mentioned in 15 abstracts, “disability” in 19, and “reproductive autonomy” in 17. Arguments were made about negative and positive eugenics with distinction from liberal eugenics and reproductive autonomy [39, 40],distinctions between choosing against disability and discrimination against disabled people [41],social justice, self-determination, dignity, and worth of the individual [42]. Additionally, some articles defended the preservation of genetic diversity to protect the relationship between humans and nature [43, 44],conditional acceptance of the child by the parent and the potential negative impact on parent–child relationship [45].

In 2001, Julian Savulescu proposed the principle of “Procreative beneficence,” arguing that parents should select among the possible children they could have, those who are expected to have the best life based on the relevant available information [46]. The term was mentioned in 15 abstracts, and “moral obligation” (of parents or society) was mentioned in 17. The debate gradually shifted to focus more on the limits to genetic selection rather than the ethics of using PGT in the first place [47, 48]. Arguments were raised, often in response to Savulescu, defending or opposing claims about parental and social moral obligation to select against diseases and disabilities or in favor of “good traits” [49–51]. Furthermore, at least 35 articles raised debates about genetic enhancement derived from hypothetical, non-proven uses of PGT to select non-medical traits [52–54].

**B2**. Fifty articles (10% of the corpus) dealt with questions of legal liability, misdiagnosis, complex selections, and deliberate selection of disabilities. They were approached by a balanced mix of study types; 38 articles (76%) were produced by authors from the U.S. (14), Netherlands (10), the U.K. (8), and Australia (6). In a nod to the previous group, these questions revolve around the legitimacy of transplanting genetically abnormal embryos, albeit for different reasons. Many articles discussed the morality of responding to parents’ desires to select disabilities, such as deafness [55], achondroplasia [56], infertility [57], or others [58]. The term “disability” was mentioned in 12 abstracts and “deafness” in 8. However, other articles discussed cases of misdiagnosis [59], or situations in which only abnormal embryos are available to implant [60, 61]. These situations became more frequently discussed with the emerging complexities stemming from the extra information new procedures yield, which may generate dilemmas regarding the selection of the right embryo [62]. This group also includes regulatory reviews and analysis of legal cases, concerning legal restrictions [17, 63], and legal responsibility or even liability (specially in tort) of parents and physicians [24, 56, 59]. The term “responsibility” appeared in 12 abstracts. Additionally, some articles explored physicians’, parents', and donors’ attitudes towards those issues [64, 65].

**B3**. A group of 30 articles examined questions about the moral status of human embryos. The most common research approach was reviews (19), followed by conceptual (Critical) analyses (11); 20 articles (66%) were written by authors from the U.K. (7), the U.S. (7), and Germany (6). The questions raised in this group were about the fate of embryos used for PGT research and of surplus embryos remaining after genetic selection [66, 67]. Other concerns include the rights of the unborn [68, 69] and the claim that an embryo should have the same dignity of a human person [70]. There were several regulatory and legal reviews in the context of human embryo status [71–74], as well as empirical studies (7 articles) capturing patients’ and physicians’ attitudes towards those morally contested fields [75, 76]. Some articles explored religious perspectives on this debate [77, 78].

**B4**. A smaller group focused on technological forecasting. Out of 16 articles, 5 were written by authors from the U.S. Some articles provided a technological review, often about new PGT possibilities, followed by an ethical discussion regarding these innovations [26, 79]. One conceptual analysis discussed ethical challenges posed by two emerging technologies: the potential capacity to produce many embryos and the ability to obtain more predictive genetic information (5719). Nevertheless, these two potentially emerging technical components were questioned. First, by other conceptual (critical) articles, which doubt the technological feasibility of using genetic selection to promote specific traits and thereby enhance human life. They mention the complex role of the genes in the context of brain, behavior, or athletic ability, and therefore criticize genetic determinism, and warn of unknown long-term health risks of PGT and ART in general [80, 81]. Second, three articles transferred knowledge from clinical data and technical experts in order to try answer such questions empirically, acknowledging that the number of available embryos for testing will, at least in the near future, remain limited and that, as much as testing and screening will enable more information, attempting to avoid all possible genetic risks might leave the physician/patient with no embryo available for transfer. Hence, the main technological limitation of PGT [82–84].

## Cluster C (red)—medical and non-medical sex selection (SS), HLA

**C1**. Eighty (15.6% of the corpus) articles dealt with the ethics and social risks of using PGT for SS, primarily for non-medical purposes. Fifty-four articles (67.5%) were written by authors from the U.S. (25), the U.K. (17), Australia (7), and Germany (5). The most common methodological approach was literature and regulatory reviews (63%), followed by, and combined with conceptual (critical) analysis, where ethical approvals and objections were raised and reviewed. It was often argued that medical interventions, like PGT for SS aimed at preventing X-linked and certain autosomal conditions affected by sex, should be strictly for medical purposes. This approach helps prevent the unnecessary medicalization of healthy individuals and wasting of limited healthcare resources [85], it was even suggested that a tax should be imposed on these elective services [86]. A common concern was the distortion of sex ratio, with the term appeared in 10 abstracts. This discussion often led to distinctions between cultures with preferences to male children and those with no specific preferences [87–89]. Religious views were also often discussed [90], as well as allegations of sexism and women discrimination resulting from PGT for SS [91, 92]. It was also claimed that using PGT for non-medical reasons will lead, on a slippery slope, towards using it to create “designer babies” [85, 93]. Some articles discussed the question of SS as an add-on to an ongoing PGT procedure [94, 95]. Throughout the period, there is an increasing use of the terms “reproductive autonomy” (16 abstracts) and “family balancing” (12 abstracts) to justify the use of PGT for SS when no social harm is done [96, 97]. Many articles reviewed laws and regulations concerning SS [93, 98], and empirical research (20 articles) included some analysis of clinical data, and mostly extraction of experts’ (physicians, ethicists, and social scientists) views through surveys, interviews, and focus groups [75, 99].

**C2**. A research niche centered on general population’s attitude and preferences towards SS. It included 29 articles, with 21 (72%) being empirical research. Fifty-two percent were written by authors from the U.S. (9) and Australia (6). Most articles were based on surveys and interviews with patients or potential patients, and they focused on two aspects: laypeople’s views and concerns regarding the social and ethical issues surrounding SS [100–102]. And motivations and gender preferences for SS in different cultures [103, 104]. Some papers suggest that there are significant preferences in the U.S. to use PGT for the selection of boys [105, 106]. Additionally, some articles analyzed media reports and online discussion forums to understand social and ethical views [107, 108].

**C3**. A group of 52 articles, accounting for 10% of the corpus, focused on the ethical review of PGT-HLA. Thirty-three (63%) articles were written by authors from the U.S. (15), U.K. (12), and Australia (6); 65% being literature and regulatory reviews, and 40% being critical analysis. The overall attitudes towards PGT-HLA were positive, with some concerns regarding a slippery slope towards “designer babies” [109]. The most discussed ethical issue was whether the procedure instrumentalizes the donor child and disrespect to its autonomy or intrinsic worth [110, 111]. One supportive argument emphasized the tie between the welfare of the future donor child and the welfare of its family as a whole, suggesting that the donor child’s welfare may ultimately be enhanced by virtue of its involvement in the shared family effort to save the life of the existing sibling [112]. Many articles also reviewed policy debates and regulations, occasionally with a comparison of different countries [109, 113, 114]. An interesting suggestion was the need to make sure that the child’s wish is not solely motivated by the therapeutic purpose [73, 74]. Empirical research (12 articles) included reports of task forces producing ethical guidelines aimed at protecting the future interests of the donor child-to-be [14, 111],analysis of real-life case studies [115]; and a few articles capturing patients’ views, attitudes, and beliefs, in a few countries, concerning practices and ethical issues of PGT-HLA [65, 116, 117].

## Cluster D (blue)—attitudes and knowledge, late-onset, counselling, and psychological impact

**D1**. A group of 51 articles (10% of the corpus) focused on the ethics, attitudes, and knowledge concerning PGT for late-onset diseases. Sixty-one percent of these articles had an empirical approach; 75% were written by authors from the U.S. (24), Netherlands (9), and the U.K. (5). The articles discussed PGT for late-onset diseases, mainly breast and ovarian cancer (more than 30 articles). They surveyed or interviewed patients and carriers about their attitudes, knowledge, awareness, and choices concerning PGT for late-onset diseases [27, 118], as well as professionals’ views on the matter [119]. Others discussed general ethical issues through reviews or conceptual (critical) analysis, with an emphasis on the severity of the condition, its treatability and the medical complications arising from it, while giving decreasing significance to the age of onset as a criterion for determining ethically acceptable applications [120–124]. Concerning the Huntington disease, mentioned in 8 abstracts, the most common concern is offering carriers the option of non-disclosing PGT. This approach enables couples to have children who are not affected by the disease while also allowing them to remain uniform about their own genetic status regarding Huntington’s [125, 126].

**D2**. Forty-eight articles analyzed patients’ attitudes and knowledge and their impact on reproductive decisions prior and after PGT. The majority, 41 (85%) were based on empirical research, and 71% were produced by authors from the U.S. (21), Germany (7), and the Netherlands (6). These articles were primarily based on surveys and interviews, with some reviews or critical analysis using previously published data. They examined the social and moral concerns of couples towards PGT from a practical perspective, specifically how their attitudes influence their decisions to pursue PGT services [65, 100, 123, 127]. Many of these articles also focused on patients’ motivations and interest in accepting or declining PGT-A as an add-on to ART [128, 129]. Additionally, a few articles analyzed the influence of media on patients’ decision-making concerning PGT [130–132].

**D3**. Twenty-six articles dealt with the counselling and informed consent process prior to PGT, from the physicians’ perspective. Nineteen (73%) used an empirical research approach. Half (13) were written by authors from the U.S. These primarily included surveys, interviews, and focus groups with physicians or patients, aimed at analyzing knowledge about PGT among different medical professionals [133], their views and strategies for counselling [134, 135], and the methods of communicating this information to patients [136, 137].

**D4**. A group of 20 articles analyzed the patient’s experience and psychological effects of PGT (the corpus included only psychology literature with ELSI or impact on patients’ decision). The majority, 13 (65%) were based on empirical research, with 60% written by authors from the U.K. (6) and the U.S. (6). These articles used surveys, interviews, and reviews of previous empirical research, to examine patient’s experience and the impact on their decision-making [138, 139]. They also evaluated psychological impact [140–142], some with a focus on PGT-A [143, 144]. Studies have found that men and women are psychologically affected, even a few years after treatment, feeling anxiety and depression, and have concerns about their relationship and about questions that children might ask concerning the procedures. Many articles emphasized the importance of providing appropriate psychological counselling.

## Cluster E (purple)—regulation and restrictions, religion, and social values

**E1**. The largest group in this cluster included 61 articles that covered national regulations. Of them, 52 articles (85%) were literature and regulatory reviews. Forty-eight percent were from the U.S. (14), Germany (9), and the U.K. (6). There were at least 20 articles of cross-national research, i.e., an author from one country conducted research about another. The majority reviewed one or several countries, asking who is or should be responsible for regulation [145], how PGT is regulated [73, 74, 146, 147], which regulatory reforms have been conducted and their impact [148, 149], or which regulatory gaps could be identified [8, 23, 150]. Some articles conducted a review and comparison of a large number of countries [151–153]. Additionally, a few articles proposed global approaches for the regulation of PGT [154, 155].

**E2**. This group includes 41 articles about the impact of religious beliefs on ethical analysis, state regulation, and people’s decision-making. There were 17 articles (41%) from the U.S., in addition to authors from 18 more countries. Fifteen articles discussed Christian views (7 Catholicism), 15—Islamic views, 12—Jewish views, 2—Buddhism, 1 – Hindu, and 1—Confucianism. The most discussed field was SS (16 articles), six articles discussed access to treatment amid religious restrictions, and at least five discussed religious beliefs concerning expanded uses of PGT and genetic enhancement. There were mainly two different types of approaches: First, more than half the articles conducted mainly literature or regulatory reviews, in addition to some conceptual (critical) analysis and empirical research based on professionals’ or clerics’ views, to discuss the impact of different religious perspective on ethical approaches and state regulation [78, 90, 156, 157]. Second, at least 14 articles conducted empirical research through interviews, surveys, analysis of media, or discussion forums, to extract and debate the impact of individuals’ religious views on their decision-making in regard to different applications of PGT [158–161].

**E3**. A small group of 24 articles dealt exclusively or partially with Cross-Border Reproductive Care (CBRC). It mainly included literature and regulatory reviews (67%) with 6 articles written by authors from the U.S., and the rest by authors from 13 other countries. Most articles focused PGT for medical indications, with 9 discussing SS and 3 with PGT-HLA. Three main issues were addressed: First, the limitations of restrictive regulation and its strong association with CBRC [162]. Many couples whose children are at risk would make a great effort to travel for PGT services [163, 164]. In the case of SS, those with the desire and financial means will travel abroad for the services [23, 165]. Second, the human rights issues, such as the exploitation of vulnerable groups by imposing a burden on public health system,the protection of reproductive and commerce freedom; the vulnerability of those with need for PGT but no financial means for CBRC [163, 164]. Third, international challenges and the need to establish a certain level of global regulation while organizing CBRC [166, 167].

## Discussion

We conducted a review of the global academic literature on the ELSI of PGT published in English from 1999 to 2019, and shown a significant increase in research output. Our keywords, content, and thematic analyses allowed us to categorize the literature into groups, identify research gaps, and explore the geographic distribution of ELSI research efforts.

Our analysis reveals a high level of geographical concentration among the countries of the corresponding authors. Between 1999 and 2019, authors from 12 countries, in the following order, U.S., U.K., Australia, Netherlands, Germany, Belgium, Canada, Israel, Italy, France, Spain, and Sweden, accounted for 86% of the research output. While research contributions from Italy, France, and Spain have increased, the share of Germany and Belgium has decreased. As illustrated in Table 2, aside from the five leading countries, no other country notably dominates any specific group of research questions. Geographical dispersion of ELSI research is an important way to produce findings that are representative and comprehensive, since local socio-cultural contexts significantly shape research framing and analysis, leading to variations between societies.

Our findings show that some issues that received significant attention from ELSI researchers do not correspond to those prevalent in the clinic. Notably, frequently debated issues, such as using PGT to select “the best” children (B1), are not clinically feasible nor practiced at this time. Others, such as deliberate selection of disabilities, PGT for sex selection (C1 and C2) and PGT-HLA (C3), are marginally used. Our findings also show that some issues that received attention are more relevant in the clinical context, including ethical attitudes and knowledge related to late-onset diseases (D1), factors influencing patients’ decision-making (D2), legal liability and misdiagnosis in PGT (B2), national regulations (E1), and the impact of religious beliefs on regulations and patients’ decision-making (E2). Finally, some critical ELSI are underexplored in the literature and we argue that they deserve greater attention, such as public versus private funding of PGT, access, financial burden (A1), the increasing use of PGT-A (A2), requests to implant affected embryos when no alternatives are available (B2), and the technological feasibility of radical scenarios (B4).

### Issues receiving ample ELSI attention, but are not clinically prevalent

ELSI researchers are often drawn to challenging and controversial ethical cases related to PGT. A significant portion, about a fifth of the entire corpus, focused on the ethics of selecting “the best” children (B1). This literature, mainly comprised of critical analysis, as an exchange between scholars from the U.S., the U.K., and Australia, delves into philosophical debates on human nature and clinical-scientific interventions meant to shape the identity of prospective children. However, findings from the group addressing technological forecasts and expectations concerning PGT (B4) suggest that the most radical selection scenarios in B1 are non-viable in clinical-scientific terms. The genetic basis of complex physical or cognitive traits is intricate and not easily selected. Furthermore, there are limits to the number of available eggs and embryos for complex selections and the production of artificial gametes remains uncertain and may cause epigenetic complications, as raised by some articles from group B2 and B4 [61, 83, 84]. If any of these radical scenarios become clinically relevant in the future, they would more likely involve germline genome editing by CRISPR rather than PGT [82].

Sex selection (SS) (C1 and C2), mainly for non-medical aims, is the second most debated issue, primarily discussed by researchers from the U.S., the U.K., Australia, and Germany. The literature acknowledges cultural distinctions between regions with male child preferences, potentially distorting sex ratios, and those with no preferences. Particularly, in China and India, selective abortion has significantly skewed sex ratios creating grave social issues [87, 88], signaling a clear social risk associated with PGT for SS. While such discussion exists, broader engagement, especially raising more insights in English from non-Western perspectives, could enrich the debate. PGT for SS also raises concerns about sexism and relates to slippery slope and enhancement arguments. Despite its controversial nature, it remains a minimally utilized procedure, prohibited in many countries, notably in Asia [151, 168], although practiced more openly in the U.S. [169]. It is usually not a primary reason for seeking ART or PGT and is often more pertinent when considered a supplementary procedure alongside other PGT applications [170, 171]. Similarly, PGT to select disabilities (B2) and PGT-HLA (C3) are marginally used clinical procedures that spark intense debates and philosophical arguments.

Though PGT for late-onset diseases may not be widely used clinically, numerous articles addressed ethical attitudes and knowledge related to these conditions (D1), highlighting their importance in the evolving PGT landscape. PGT-P for polygenic conditions, along with the already prevalent use of PGT-A for aneuploidy screening, may broaden PGT application to more ART cycles. While a smaller percentage of the population is affected by monogenic conditions or hereditary chromosomal abnormalities, many are susceptible to polygenic conditions such as diabetes, cancer, and cardiovascular diseases. Aiming on late-onset diseases could contribute to more widespread adoption of PGT in the future, in the context of polygenic conditions, and hence deserves the ELSI attention it is receiving.

It is understandable that many ELSI scholars, often trained in humanities and social sciences, find extreme scenarios and “hard cases” conceptually fascinating and philosophically challenging [44, 46]. Techniques that are not currently offered, or even not possible, may still raise theoretically interesting issues that have general implications for human identity, family dynamics, disability rights, fear of eugenic consequences, and the overall character of society. In that sense, ELSI work on issues that are not relevant to clinical practice or that are only marginally used in the clinic is still valuable in the insights it offers. However, we should keep in mind that research resources and intellectual energy are also limited. When about 40% of the literature is dedicated to issues that scarcely affect patients in reality, other issues—that affect them much often—remain underexplored, as we discuss below.

Furthermore, often novel and emerging technologies—and their most controversial uses—that intrigue ELSI researchers actually exacerbate disparities and widen the gaps in terms of equity of access. IVF itself is a case in point. As an intervention that is not publicly funded in most countries, it creates clinical and social disparities in terms of access to fertility care. PGT in general is another case in point. As such, the more established and less controversial uses of PGT (e.g., for selection against serious monogenic diseases and chromosomal abnormalities) are the ones that may receive public funding sooner [18, 172], compared with more rare and ethically controversial uses, that will probably remain an out-of-pocket service available only to those who have resources. As such, the heightened ELSI attention given to extreme scenarios is dedicated to uses that will only benefit the privileged few, at the expense of a deeper discussion of issues of equity of access and the overall fairness of the fertility industry.

### Issues receiving ample ELSI attention, and are clinically prevalent

Our findings show that certain clinically relevant issues have been extensively covered in the ELSI literature, with a notable focus on comparative analysis of regulations worldwide (E1). This research involved authors from 18 countries, addressing regulations in at least 29 countries, revealing diverse regulatory models and the impact of religious laws (E2). Researchers compare jurisdictions and highlight challenges arising from differences, particularly in cross-border reproductive care (E3). With some procedures banned in certain countries while open to market forces in others, it is crucial to consider the implications of cross-border reproductive care for SS, also as an indicator for future applications of PGT or gene editing.

A substantial number of articles investigated attitudes and knowledge (D2) and religious beliefs (E2), and their influence on patients’ decision-making. Most articles on attitudes and knowledge originated from the U.S., Germany, and the Netherlands, while those about religious beliefs covered a broader geographical range. These primarily empirical articles shed light on individual perspectives, cultural factors, and emotional barriers related to PGT. This attention dedicated to cultural perspectives is important, considering the critical role such values play in patients’ experiences and preferences in the context of fertility care in general, and PGT in particular.

### Issues that are clinically prevalent and require more ELSI attention

Some issues relevant in the clinical context received inadequate ELSI attention, particularly public and private funding of PGT, equitable access, and the financial burden on patients (A1). The importance of PGT for families with hereditary conditions should be considered, since in most countries PGT is privately funded and there is an insufficient ELSI research on cost, access, financial impact on families, and cost-effectiveness (A2). The issue of cost-effectiveness has ELSI implications on two distinct levels. First, the use of PGT-A as add-on to increase the chances of a viable pregnancy, which is currently hotly contested from a clinical standpoint [61, 173, 174] and from an ELSI perspective, since clinics have an inherent conflict of interest in offering pricy services that enhance their profits, with unclear effectiveness for patients’ clinical outcomes. Recently, PGT-A has become part of a high percentage of cycles in the U.S. and of an increasing number in Europe, imposing significant costs on patients with unclear effectiveness (A2) [175, 176], ESHRE, 2022. Researchers could further examine clinic’s marketing strategies of this service. One cost-effectiveness analysis found PGT-A to be cost-effective in specific clinical settings and population groups, improving with female age [177]. In contrast, another analysis concluded that, from the perspective of healthcare providers in China, embryo selection with PGT-A is not suitable for routine applications due to the cumulative live birth rate (CLBR) and high costs [178].

The ELSI issues raised by the cost-effectiveness of PGT-A are distinct from those raised by PGT to select against conditions that will be costly to treat over the life of the child/adult. The cost of raising children with conditions tested by PGT is thus another issue that could be the topic of further ELSI investigation. While addressing these costs in the context of prenatal testing involves potential pressure on pregnant people to terminate and eugenic messages [179], in the context of PGT, selecting against diseases may be seen as less ethically problematic, since there is no pregnancy yet and usually not all embryos can be implanted, so selection has to occur in any case. Some have argued that the selection of certain embryos is in and of itself eugenic [180], but overall, selection through PGT is seen as less ethically controversial than selection through pregnancy termination.

The ELSI implications of cost–benefit analysis in terms of reducing the burden of cost of care over the life of the child are underexplored in the literature, and we only identified two articles (from A1) that discuss this in relation to caring for cystic fibrosis patients. Researchers could further examine costs such as the daily effort of parents caring for sick children, and the long-term impact on parents’ health, relationship, careers, and other children, which were not adequately explored in the ELSI literature.

Other underexplored issues involve emergent concerns in reproductive medicine center on the dilemma of embryo transfer when only affected embryos are available, and no alternative options available. This predicament is becoming increasingly prevalent due to the intricate insights garnered from recent procedural advancements [61, 62]. Enhanced PGT screening, in particular, offers more granular data on each embryo, frequently uncovering a greater prevalence of abnormalities. Consequently, this leaves patients and clinicians with only affected embryos for potential transfer. Patient desires to implant such embryos introduce complex ELSI issues, highlighting the tension between patients’ reproductive autonomy and clinicians’ ethical commitment to “do no harm.” Notably, these ethical intricacies saw growing attention in academic circles, especially during 2018–2019 in the researched corpus.

### Limitations

Our analysis has several methodological limitations. First, our selection and cleaning process inevitably involves a degree of subjectivity. Complex selection criteria and author-selected terms may have led to both false-positives and false-negatives. Our efforts to exclude medical-technical articles might have resulted in a scarcity of ELSI research driven by clinicians or performed in collaboration with them, potentially leading to false-negative errors, particularly missing some cost-effectiveness and clinical outcome articles if focused more on medical issues and less on ELSI. Second, we did not update our data beyond 2019 due to the intricate challenges involved in screening and cleaning the database. Given that ELSI research on PGT is a continuously evolving field, this represents a modest constraint on its present relevance. Third, while geographic distribution is not a core element of this paper, it is worth noting that associating an article with countries based solely on the corresponding author’s affiliation and the abstract’s content may cause some mistakes, despite our efforts to manually identify and correct them. Fourth, the low share of psychology articles should be considered in light of our database selection, which only includes articles with ELSI or impact on patients’ decision-making and not all psychological research about fertility care. Finally, it is worth mentioning our inability to conduct a systematic literature review or meta-analysis due to the large variety of approaches, methodologies, and prevalence of conceptual papers. Similarly, we identified only 1% of meta-analyses in the ELSI of PGT literature. There may be room to develop innovative methods to address these challenges.

## Conclusion

In examining the ELSI of PGT literature, we found some disparity between the prevalent issues discussed in the literature and those patients encounter in real-world clinical settings. While some scenarios have received much attention, such as PGT to select “the best” children, deliberate selection of disabilities, PGT for sex selection and PGT-HLA, there are pressing matters related to reproductive justice, notably issues of equity of access, economic burden on patients, and cost-effectiveness, that require further exploration. Particularly concerning is the role of PGT-A in clinical settings, as clinics navigate the tensions surrounding the offer of potentially high-cost interventions with debated effectiveness, the inherent conflicts of interest become increasingly prominent. Our findings also show that the evolving challenges regarding expanded use of PGT to screen for additional conditions and traits deserve more attention. Ethical dilemmas emerge when only affected embryos are identified, raising tensions between the reproductive autonomy of patients and clinicians’ sense of professional responsibility. In light of these findings, we call the ELSI research community to dedicate attention to underexplored issues, to close these gaps, and to offer guidance to patients, clinicians, and policymakers.

Our study emphasizes the need for a closer collaboration between clinicians, economists, and policymakers on one hand, and ELSI scholars on the other. As technologies evolve and our genetic insights become richer and more refined, it is beneficial for humanities and social science researchers to remain closely aligned with scientific and technological advancements. This would ensure that conjectured scenarios are based on realistic parameters, allowing ELSI researchers to address the real and pressing concerns of patients and clinicians, and to make more informed predictions about potential outcomes. A focus on demystifying complex laboratory processes for the general public can also bridge the understanding gap, helping society comprehend the benefits and potential risks associated with PGT technologies.

Addressing the ELSI of PGT requires a comprehensive, patient-centered approach that truly captures the challenges patients and clinicians face, aiming to provide a balanced and constructive perspective in the evolving ART discourse.

## Data Availability

The data that support the findings of this study are available on request from the corresponding author.

## Appendix 1

**Table 3:** Inclusion and exclusion criteria for articles in the review

Inclusion criteria	Exclusion criteria
All articles concerning all sorts of ART and assisted insemination (AI) which are dealing either: • Fully or partially with demographic (activity reports and registries, geographical and socioeconomic patterns of usage), ELSI, economic, regulations, anthropological, and political issues. OR • With value-based approaches, attitudes, opinions, expectations, and preferences. OR • With education, knowledge, and empowerment of decision-making among patients and donors. OR • With psychology and quality of life issues, raising ELSI or affecting government policymaking and patients’ decision-making	Articles which focus on either: • Clinical methods/outcome or technical issues with no relation to ELSI or social sciences. OR • Physicians’ professional approaches, opinions, preferences, education, and knowledge concerning clinical decision-making of technical character. OR • Psychology/quality of life outcomes by way of empirical research with no ELSI or impact on patients’ decision-making
Rejection criteria: Articles dealing exclusively with 1. Animal research. 2. Treatment outcome, unless measured in terms of population and socioeconomic characteristics (i.e., national registries). 3. Clinical policies on a local level (in contrast to national/regional policies). 4. Clinical outcome and performance of technologies and protocols. 5. Processes within hospitals and clinics. 6. Prenatal testing and selection. 7. Therapeutics (non-ART) uses of stem-cell research. 8. Clinical trials or reports in psychiatry/psychology with no ELSI or impact on patients’ decision-making	

## Appendix 2

**Table 4:** MeSH terms and keywords

Group A – Inclusion of ART terms	MeSH terms (for PubMed) (28)
	Reproductive Techniques, Assisted • Donor Conception • Embryo Transfer • Single Embryo Transfer • Fertility Preservation • Fertilization in Vitro • Mitochondrial Replacement Therapy • Sperm Injections, Intracytoplasmic • Gamete Intrafallopian Transfer • Insemination, Artificial • Insemination, Artificial, Heterologous • Insemination, Artificial, Homologous • Oocyte Donation • Oocyte Retrieval • Posthumous Conception • Embryo Disposition • Sperm Banks • Surrogate Mothers • Preimplantation Diagnosis • Sex Preselection • Gene Editing • Genome Editing • Genetic Enhancement • Adult Germline Stem Cells • Germ Cells • Ovum • Oocytes • Embryo Research • Research Embryo Creation
	Keywords (for WoS and Scopus) (38)
	assisted reprod* • assisted procreat* • reproductive techn* • in vitro fertili* • intracytoplasmic sperm injection • In vitro gametogenesis • egg don* • oocyte don* • sperm don* • embryo don* • donor eggs • donor conception • fertility preservation • oocyte cryopreserv* • egg freez* • sperm cryopreserv* • embryo cryopreserv* • oncofertility • leftover embryos • surplus embryos • frozen embryos • preimplantation genetic • reproductive genetic* • germline engineering • germline gene editing • germline gene modification • germline genetic modification • mitochondrial replacement • mitochondrial don* • surrogacy • surrogate mother • gestational carrier • uterus transplantation • artificial insemination • donor insemination • posthumous insemination • posthumous conception • posthumous reproduct*
Group B – Inclusion of terms from disciplines of humanities and social sciences	MeSH terms (for PubMed) (23)
	Disability Evaluation • Anthropology • Demography • Economics • Forecasting • Policy • Private Sector • Public Sector • Sociology • Work-Life Balance • Education • Stakeholder Participation • History • Knowledge • Philosophy • Religion • Disabled Persons • Vulnerable Populations • Population Characteristics • Health Care Economics and Organizations • Health Services Administration • Health Care Quality, Access, and Evaluation • Attitude
	Keywords (for WoS and Scopus) (28)
	*ethic* • *access* • anonym* • attitude* • perception • *consent* • market* • crossborder • disclos* • eugen* • *identity • justi* • educat* • law* • legis* • legal* • moral* • inmoral • policy* • politic* • govern* • sex select* • touris* • view* • autonom* • desire* • vulnerab* • relatedness
Group C – Exclusion of medical-technical terms	MeSH terms (for PubMed) (45)
	Ovulation Induction • Neoplasms • Pregnancy Complications • Follicle Stimulating Hormone • Breast Neoplasms • Odds Ratio • Sperm Motility • Prognosis • Semen Analysis • Congenital Abnormalities • Gonadotropin-Releasing Hormone • Ovarian Hyperstimulation Syndrome • Polycystic Ovary Syndrome • Pedigree • Recombinant Proteins • Clomiphene • Zygote • Mice • Reproducibility of Results • Ovarian Follicle • Chorionic Gonadotropin • Sperm Retrieval • Ovarian Neoplasms • Testis • Gonadotropins • Estradiol • Randomized Controlled Trials as Topic • Oligospermia • In Vitro Oocyte Maturation Techniques • Superovulation • Zygote Intrafallopian Transfer • Polar Bodies • Zona Pellucida • Sperm Head • Acrosome • Sperm Tail • Spermatids • Spermatocytes • Spermatogonia • Embryonic Germ Cells • Plants • Case–Control Studies • Botany • Agriculture • Clinical Trials as Topic
	Keywords (for WoS and Scopus) (38)
	ovulation Induction • neoplasms • follicle stimulating hormone • odds ratio • sperm motility • semen analysis • congenital abnormalities • gonadotropin • ovarian hyperstimulation • polycystic ovary • pedigree • recombinant proteins • clomiphene • zygote • ovarian follicle • testis • estradiol • randomized controlled trials • controlled trials • clinical trials • controlled clinical trial • validation study • polar bodies • zona pellucida • sperm head • acrosome • sperm tail • spermatids • spermatocytes • spermatogonia • embryonic germ cells • plants • case control • botany • agriculture • oligospermia • oocyte maturation • superovulation

*Zero or more characters

## Footnotes

1 Percentage of transfers of at least one embryo with PGT.

2 Please note that a fully detailed description of the methods used to collect the corpus can be found in “Mapping Ethical, Legal, & Social Implications (ELSI) of Assisted Reproductive Technologies” (Alon et al., 2023).

3 To lowerize and tokenize, we used https://tedboy.github.io/nlps/generated/generated/gensim.utils.simple_preprocess.html

4 In order to produce a readable map in VOSviewer, the number of items (keywords) must be reduced. For the database, 9 was the largest number of repetitions in which at least one keyword from each of the 506 paper was represented.
